# Effect of sulfasalazine on human neuroblastoma: analysis of sepiapterin reductase (SPR) as a new therapeutic target

**DOI:** 10.1186/s12885-015-1447-y

**Published:** 2015-06-21

**Authors:** Lisette P. Yco, Dirk Geerts, Gabor Mocz, Jan Koster, André S. Bachmann

**Affiliations:** 1 Department of Pediatrics and Human Development, College of Human Medicine, Michigan State University, 301 Michigan Street, NE, Grand Rapids, MI 49503 USA; 2 Department of Pharmaceutical Sciences, The Daniel K. Inouye College of Pharmacy, University of Hawaii at Hilo, Hilo, HI 96720 USA; 3 Department of Molecular Biosciences and Bioengineering, College of Tropical Agriculture and Human Resources, University of Hawaii at Manoa, Honolulu, HI 96822 USA; 4 Department of Pediatric Oncology/Hematology, Sophia Children’s Hospital, Erasmus University Medical Center, Rotterdam, GE 3015 The Netherlands; 5 Pacific Biosciences Research Center, University of Hawaii at Manoa, Honolulu, HI 96822 USA; 6 Department of Oncogenomics, Academic Medical Center, University of Amsterdam, Amsterdam, AZ 1105 The Netherlands

**Keywords:** Drug synergism DFMO, Molecular docking, Neuroblastoma, SPR, Sulfasalazine

## Abstract

**Background:**

Neuroblastoma (NB) is an aggressive childhood malignancy in children up to 5 years of age. High-stage tumors frequently relapse even after aggressive multimodal treatment, and then show therapy resistance, typically resulting in patient death. New molecular-targeted compounds that effectively suppress tumor growth and prevent relapse with more efficacy are urgently needed. We and others previously showed that polyamines (PA) like spermidine and spermine are essential for NB tumorigenesis and that DFMO, an inhibitor of the key PA synthesis gene product ODC, is effective both *in vitro* and *in vivo*, securing its evaluation in NB clinical trials. To find additional compounds interfering with PA biosynthesis, we tested sulfasalazine (SSZ), an FDA-approved salicylate-based anti-inflammatory and immune-modulatory drug, recently identified to inhibit sepiapterin reductase (SPR). We earlier presented evidence for a physical interaction between ODC and SPR and we showed that RNAi-mediated knockdown of SPR expression significantly reduced native ODC enzyme activity and impeded NB cell proliferation.

**Methods:**

Human NB mRNA expression datasets in the public domain were analyzed using the R2 platform. Cell viability, isobologram, and combination index analyses as a result of SSZ treatment with our without DFMO were carried out in NB cell cultures. Molecular protein-ligand docking was achieved using the GRAMM algorithm. Statistical analyses were performed with the Kruskal-Wallis test, 2log Pearson test, and Student’s *t* test.

**Results:**

In this study, we show the clinical relevance of SPR in human NB tumors. We found that high SPR expression is significantly correlated to unfavorable NB characteristics like high age at diagnosis, *MYCN* amplification, and high INSS stage. SSZ inhibits the growth of NB cells *in vitro*, presumably due to the inhibition of SPR as predicted by computational docking of SSZ into SPR. Importantly, the combination of SSZ with DFMO produces synergistic antiproliferative effects *in vitro*.

**Conclusions:**

The results suggest the use of SSZ in combination with DFMO for further experiments, and possible prioritization as a novel therapy for the treatment of NB patients.

## Background

Neuroblastoma (NB) is a childhood cancer that mainly affects children up to 5 years of age [1–6]. NB is risk-stratified according to patient age at diagnosis, disease stage (INSS stages 1–4 and 4 s), and common genetic aberrations like MYCN oncogene amplification. This NB classification is used to determine the treatment regimen, and is effective in predicting patient survival. Survival rates range from > 90 % for low- to < 50 % for high-risk NB [7–10]. Patients that suffer from high-risk NB, especially those with tumor MYCN gene amplification, show incomplete response to aggressive, multimodal therapy and often relapse and ultimately die [1–6]. While considerable progress in survival was attained by optimizing conventional interventions like chemotherapy, radiation, and bone marrow transplantation, it is now widely accepted that a therapeutic plateau has been reached. Increased treatment intensification is not considered likely to improve patient outcome in high-risk NB [11, 12]. Instead, the reduction of the grave treatment complications by fine-tuning risk-adapted therapy, and the development of more effectual, more specific, and less harmful molecular targeted drugs are currently viewed as the most important policies.

We and others have studied the polyamine (PA) biosynthetic pathway and its enzymes as novel targets in NB. High PA levels increase tumor cell proliferation and survival in NB and many other cancer types [13–17]. For NB, we have published that PA depletion upon addition of alpha-difluoromethylornitine (DFMO), which inhibits the key PA biosynthesis enzyme ornithine decarboxylase (ODC), readily decreases cell proliferation by activating the p27^Kip1^/retinoblastoma (Rb) signaling axis and by inducing cell cycle arrest in the G_1_ phase [18, 19]. We also showed that *S*-adenosylmethionine decarboxylase (AdoMetDC, also known as SAMDC or AMD) is important for PA production in NB [20] and that PAs contribute to NB cell migration and metastasis [21]. In addition, we assessed the role of deoxyhypusine synthase (DHPS) that uses spermidine as a substrate for post-translational activation/hypusination of eukaryotic initiation factor 5A (eIF5A), and found that its inhibition by *N*^*1*^-guanyl-1,7-diaminoheptane (GC7) had a p21^Cip1^/Rb-mediated negative effect on NB cell proliferation [22].

Importantly, DFMO was also effective *in vivo* in both human NB tumor cell xenografts in mice and the transgenic TH-MYCN NB mouse model [23–25]. Considering its excellent safety profile and its successful use in human patients in combating trypanosomiasis (or African sleeping sickness disease), we re-targeted DFMO for NB treatment, advancing the drug through the Neuroblastoma and Medulloblastoma Translational Research Consortium (NMTRC) into multicenter phase I [26] and phase II (ongoing) clinical studies [27, 28].

**Fig. 1:**
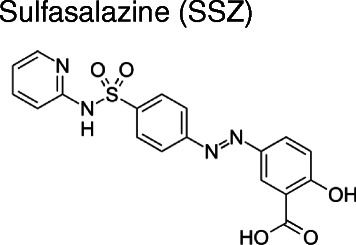
Structure of Sulfasalazine (SSZ). SSZ is an amino-salicylate, specifically 5-((4- (2- Pyridylsulfamoyl) phenyl)azo) salicylic acid (systemic name: 2-hydroxy-5-[(E)-2-{4-[(pyridin-2-yl)sulfamoyl]phenyl}diazen-1-yl]benzoic acid), with a molecular mass of 398.394 g/mol. SSZ was developed in the 1950’s to treat rheumatoid arthritis and is also indicated for the use in ulcerative cholitis and Crohn’s disease. SSZ is commercially distributed under the brand names Azulfidine, Salazopyrin and Sulazine

We have previously shown that the combination of DFMO with PA uptake inhibitor AMXT-1501 was synergistic *in vitro* [29]. In an attempt to find additional compounds interfering with the PA biosynthesis pathway, we tested sulfasalazine (SSZ), a well-documented, FDA-approved salicylate-based anti-inflammatory and immune-modulatory drug (Fig. 1). SSZ is used to treat bowel inflammation in patients with ulcerative colitis and Crohn’s disease and also indicated for use in rheumatoid arthritis. SSZ has recently been identified to inhibit sepiapterin reductase (SPR), an important enzyme in the biosynthesis of tetrahydrobiopterin (BH4) [30, 31]. BH4 is an essential cofactor in the production of serotonin, dopamine, epinephrine, norepinephrine, and nitric oxide synthase (NOS).

We earlier presented evidence for a physical interaction between ODC and SPR and we showed that RNAi-mediated knockdown of SPR expression significantly reduced native ODC enzyme activity and impeded the proliferation of NB cells, demonstrating the biological relevance of this novel interaction [32]. This current study is the first report on the cellular effects of SSZ on NB tumor cells, presumably due to the inhibition of SPR as predicted by computational docking of SSZ into SPR. We further demonstrate the clinical relevance of SPR in human NB tumors and show that the combination of SSZ with DFMO produces synergistic antiproliferative effects, suggesting the use of SSZ/DFMO combination therapies in NB patients.

## Results

### SPR mRNA expression in NB

**Fig. 2:**
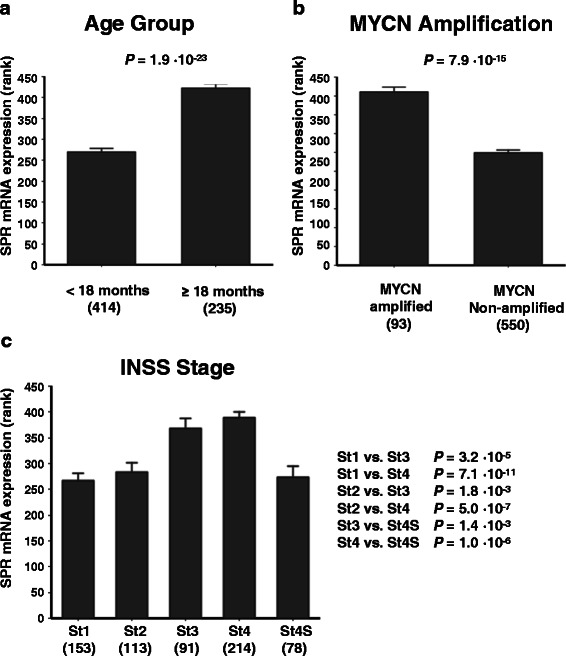
SPR mRNA expression correlation with NB clinical parameters. Differential expression of SPR mRNA expression in the Kocak-649 cohort upon separation of patient samples into clinically important groups. (**a**) SPR expression is significantly higher in older than in younger patients (age at diagnosis ≥18 months versus <18 months; *P* = 1.9 · 10^−23^), (**b**) SPR expression is significantly higher in patients with than in patients without tumor MYCN gene amplification (*P* = 7.9 · 10^−15^), and (**c**) SPR expression is significantly higher in high than in low stage tumors (INSS stage 3 and 4 versus stage 1, 2, and 4S; various *P* < 0.05). For all three parameters, SPR expression is highest in the poor outcome group. Statistical analysis was performed using the non-parametric Kruskal-Wallis tests

**Fig. 3:**
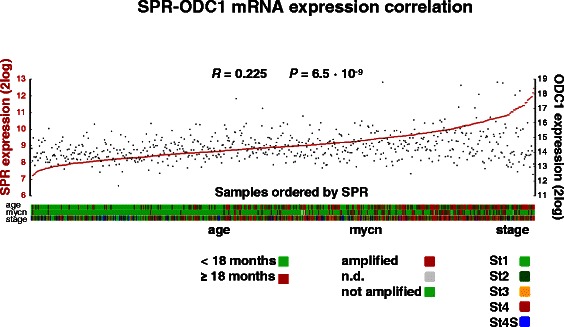
SPR expression correlation with ODC expression in NB. SPR and ODC mRNA expression correlation in the Kocak-649 NB cohort: visual representation of SPR and ODC expression in all 649 NB tumor samples, ranked horizontally from left to right according to their SPR expression. SPR and ODC (2log) expression values for each sample are visualized with red circles and black rectangles, respectively. The correlation between SPR and ODC expression is r = 0.225, with a *P* value of 6.5 · 10^−9^ (2log Pearson). Symbols representing the clinical values of the tumor samples: age at diagnosis, MYCN amplification, and INSS stage, are listed below the graph, together with their legend

We have previously reported on the role of SPR in NB proliferation [32], where we demonstrated a deleterious effect of RNAi-mediated SPR expression knockdown in the MYCN2 NB cell line. We also showed that high SPR mRNA expression was correlated to poor patient prognosis in Kaplan-Meier analysis in the Versteeg-88 NB dataset in the public domain. We now present SPR mRNA expression analysis on all 12 NB cohorts in the public domain (Table 1). We find that high SPR expression is significantly correlated in all four NB cohorts annotated for patient survival and/or prognosis. While in our previous study [32] we could only show a trend for a correlation between SPR expression and tumor MYCN gene amplification in the Versteeg-88 set (*P* = 0.06), we can now state that SPR expression is significantly higher in patients with tumor MYCN gene amplification in 6 of 8 datasets with MYCN amplification annotation. Considering the different compositions of these datasets with respect to patient age, MYCN amplification, and INSS stage, together with the different array platforms used for the generation of these data, this is a very robust finding. In Fig. 2, we show the results for the largest NB cohort in the public domain, the Kocak-649 dataset. Although this dataset does not contain survival data, the correlations between SPR expression and three important clinical NB parameters are highly significant (Fig. 2, a-c): age at diagnosis (*P* = 1.9 · 10^−23^, MYCN tumor amplification (*P* = 7.9 · 10^−15^, and INSS stage (various *P* values < 0.05). In addition, the Kocak-649 dataset shows a significant correlation between SPR and ODC mRNA expression (Fig. 3, R = 0.225, *P* = 6.5 · 10^−9^). This association, although highly significant, has a relatively low R value. However, since we previously found a similar association (R = 0.289, *P* = 6.2 · 10^−3^) in the Versteeg-88 cohort [32], we felt strengthened in our argument that this correlation is meaningful.

**Table 1:** SPR mRNA correlations in public NB mRNA expression datasets

Dataset		SPR mRNA expression correlations		Micro-array data	
Name	Samples	Survival/prognosis	MYCN amplification	Array Type	GSE
Delattre	64	n.d.	positive (6.8 • 10^-6^)	Affymetrix HG-U133 Plus 2.0	12460
Hiyama	51	negative (0.02)	positive (2.8 • 10^-3^)	Affymetrix HG-U133 Plus 2.0	16237
Jagannathan	100	negative (0.02)	positive (1.7 • 10^-3^)	Illumina Human WG 6V2	19274
Kocak	649	n.d.	positive (7.9 • 10^-15^)	Agilent Human 44K Oligo	45547
Łastowska	30	n.d.	positive (2.6 • 10^-4^)	Affymetrix HG-U133 Plus 2.0	13136
Maris	101	n.d.	n.s.	Affymetrix HG-U95A	3960
Seeger	117	negative (1.4 • 10^-4^)	n.d.	Affymetrix HG-U133A	3446
Versteeg	88	negative (0.02)	n.s.	Affymetrix HG-U133 Plus 2.0	16476
Zhang	498	negative (2.1 • 10^-6^)	positive (4.6 • 10^-4^)	Agilent Human 44K Oligo	49710

**Legend:** The Albino-28 (GSE7529), Khan-47 (GSE27608), and Seeger-102 (GSE3446) do not contain sufficient clinical data and were not analyzed. Data were analyzed as described in the Materials and Methods. The first two columns represent name and sample size of the dataset. The two central columns show the results of SPR mRNA expression correlation analyses: with survival and/or prognosis, and with MYCN amplification. Negative or positive in the two central columns means that SPR mRNA expression correlates negative or positive with survival/good prognosis and MYCN amplification, respectively (outcomes of Kruskal-Wallis correlation tests, the number in parentheses is the *P* value, n.s. means not significant, n.d. means not determined (data not present in the dataset)). Kocak-649 and Zhang-498 contain some common samples. The last two columns list Array type and GEO GSE number on the NCBI GEO website where full data are available

These results show that SPR mRNA expression is highest in all NB clinical groups with poor outcome: high age at diagnosis, tumors with MYCN oncogene amplification, and patients with high INSS tumor stage. Its expression pattern therefore resembles that of ODC, and indeed we found a tentative correlation between SPR and ODC expression. Together, these results prompted us to investigate the specific targeting of SPR alone or together with targeting of ODC as novel NB therapy.

### The effect of Sulfasalazine (SSZ) treatment on NB cell proliferation and survival

**Fig. 4:**
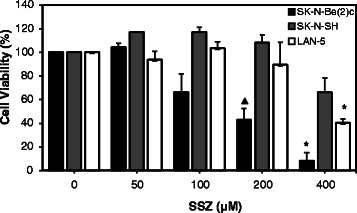
Effect of Sulfasalazine (SSZ) on the viability of NB cells using the MTS cell viability assay. NB cell lines SK-N-Be(2)c, SK-N-SH, and LAN-5 were treated with increasing concentrations of SSZ for 48 hours. Dose-dependent inhibition of cell viability was observed. Statistically significant differences between values obtained from DMSO-treated control cells and SSZ-treated cells are indicated with an asterisk (*P < 0.05) or solid triangle (*▲P < 0.005*). Data represent the average of three independent experiments (n = 3); bars, mean ± SEM

A recent study by Chidley *et al.* revealed that SSZ blocks BH4 biosynthesis through inhibition of SPR [30]. To examine the inhibitory effects of SSZ in NB cells, we treated SK-N-Be(2)c, SK-N-SH, and LAN-5 cells with increasing concentrations of SSZ (0–400 μM) and measured cell viability 48 h after treatment. As shown in Fig. 4, SSZ decreased the cell viability of all three NB cell lines in a dose-dependent manner. We did not observe overt apoptosis (data not shown), suggesting that SSZ inhibits cell proliferation of NB cells without cytotoxic effects.

To investigate potential signaling molecules and pathways involved in SSZ-mediated cell death, we tested the expression levels of several proteins that regulate cell proliferation, including p27^Kip1^, retinoblastoma tumor suppressor protein Rb, Akt/PKB, and p44/42 MAPK (Erk1/2). Western blot analysis did not reveal any significant protein expression differences between SSZ-treated and untreated NB cells (data not shown), suggesting that additional, alternative signaling pathways are activated by SSZ.

### Computational modeling and docking of SSZ into SPR

To examine if SPR binds SSZ, we performed computational docking simulations. SSZ is an amino-salicylate, specifically 5-((4- (2- Pyridylsulfamoyl) phenyl)azo) salicylic acid (Fig. 1). SSZ has one canonical conformer with an MMFF94-minimized (Merck Molecular Force Field) energy of 83.9 kcal/mol, which was used in the docking simulations [33]. Under physiological conditions the molecule carries a negative charge which may have a role in the interaction with the receptor.

The human SPR crystal structure is available in complex with NADP+ in a hexameric assembly (unpublished data, PDB: 1Z6Z). This biologically active, functional form of SPR exists as a dimer and has 2-fold (180°) rotational symmetry. The SPR monomer is an alpha and beta (a/b) class protein with a 3-layer (aba) sandwich architecture and Rossmann fold topology, and it contains an NADP- binding Rossmann-like domain [34].

We explored feasible binding modes both for the SPR monomer and the dimer. The docking computations were carried out on each binding mode by geometric complementarity and semi-flexible docking to allow for inherent receptor flexibility. From each computation, the 50 lowest energy-docking positions were saved for further analysis. The presumed SSZ-binding sites were ranked by conservation score, specifically by the frequency of occurrence of a residue in a contact surface. The contact surface was delimited as an area consisting of the residues inside a 3.6 Å radius of the ligand.

Based on the conservation scores of all the residues, we identified the main binding location within the NADP-binding Rossmann-like domain. A consensus of five binding regions constituted the receptor pocket comprising residues Gly11, Ser13, Arg14, Phe16 (Region 1), Ala38, Arg39 (Region 2), Asn97, Ala98, Gly99, Ser100 (Region 3), Tyr167 (Region 4), and Leu198, Thr200, Met202 (Region 5). Thus, the binding pocket appeared to contain 2 basic polar residues, 5 neutral polar residues, and 7 neutral non-polar residues. Due to the presence of 2 arginine residues, the site has a basic, positively charged character which may be essential for SSZ binding. Most or all of SSZ exists in a non-protonated, negatively charged state at neutral pH, as the acidic pK_a_ of carboxylic acid is 2.3 and the pK_a_ of the sulfonamide nitrogen is 6.5, *i.e.* less than half-protonated at pH 7.0 [35].

The same residues listed above are involved in NADP+ binding, but the complete NADP+ binding site extends beyond these residues (Table 2). The monomeric or dimeric state of SPR did not affect the location of the SSZ binding site in the simulations, indicating that dimerization does not directly block the access of ligand to the receptor. Table 2 also lists the dimer interface residues. Indeed, the interface residues do not share common elements with the SSZ/NADPH+ binding pocket. Only Tyr167, which is part of both ligand sites, is found in the vicinity of an interface residue, *i.e.* Cys168.

**Table 2:** Amino acid residues at the binding sites of SPR-SSZ, SPR-NADP+, and SPR-SPR complexes

SSZ	NADP+	SPR Dimer
Pocket	Pocket	Interface
Gly11	Gly11	-
Ser13	Ser13	-
Arg14	Arg14	-
	Gly15	-
Phe16	Phe16	-
Ala38	-	-
Arg39	Arg39	-
-	Asn40	-
-	Ala65	-
-	Asp66	-
-	Leu67	-
-	-	Glu70
Asn97	Asn97	-
Ala98	-	-
Gly99	-	-
Ser100	-	-
-	-	Gly107
-	-	Phe108
-	-	Val109
-	-	Asp110
-	-	Leu111
-	-	Ser114
-	-	Val117
-	-	Asn118
-	-	Trp121
-	-	Ala122
-	Leu123	-
-	-	Thr126
-	-	Leu129
-	-	Ser133
-	-	Lys137
-	Ile152	-
-	Ser153	-
-	-	Pro160
-	-	Phe161
-	-	Lys162
-	-	Gly163
-	-	Ala165
Tyr167	Tyr167	-
-	-	Cys168
-	-	Ala169
-	Lys171	-
-	-	Ala173
-	-	Met176
-	-	Leu177
-	-	Val180
-	-	Leu181
-	-	Leu183
-	-	Glu184
-	Pro195	-
-	Gly196	-
-	Pro197	-
Leu198	Leu198	-
Thr200	Thr200	-
Met202	Met202	-
-	Gln203	-

Cutoff distance: 3.6 Angstrom

**Fig. 5:**
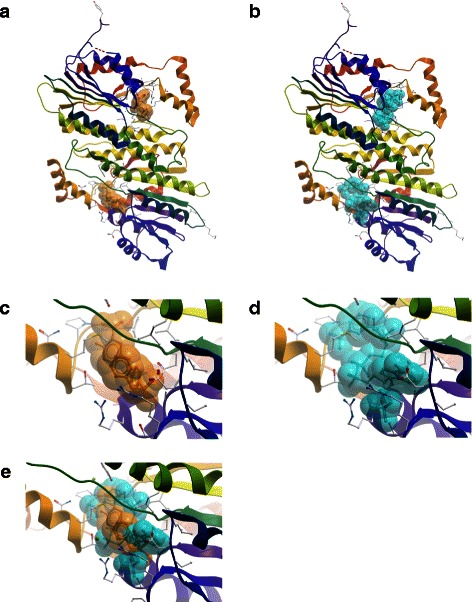
Binding of SSZ to SPR. (**a**) SPR dimer front view (C2 axis). Both chains bind SSZ independently. (**b**) SPR dimer in complex with NADP+. (**c**) SPR monomer close-up front view of the SSZ binding pocket: (**d**) SPR monomer close-up front view of the NADP+ binding pocket. (**e**) Overlay view of SSZ and NADP+ binding sites. The two binding sites overlap upon 3D alignment of the SPR protein chains. The amino acid residues involved in SSZ and NADP binding are listed in Table 2. Color scheme for the molecular constituents: Protein chain ribbon - rainbow spectrum from N-terminus (blue) to C-terminus (red); SSZ space fill – amber; NADP+ spacefill – cyan

Figure 5 shows the binding of SSZ to SPR monomer and dimer, respectively. Both chains were found to simultaneously bind ligands in the dimer. While the SSZ site is close to the N-terminus in the primary structure, it appears near the middle of the protein in the 3D fold. The binding pocket is not in very close contact with the dimerization interface and only a few side chains project into the joint neighborhood. The figure also shows the NADP+ binding site of SPR in side-by-side comparison and overlay mode with SSZ. The superimposition of the ligands clearly illustrates that the two binding sites are essentially the same. The geometric center of SSZ and NADP+ is separated only by about 0.5 Å from each other in the superimposed binding pockets. Thus, from Fig. 5 and Table 2 it appears that the binding site for SSZ coincides with the region previously identified in NADP+ binding in the X-ray structure. As a consequence, this could help elucidate the interaction between SSZ and SPR in *in vitro* and *in vivo* studies.

### Synergism of SSZ and DFMO combination treatment in NB cells

**Fig. 6:**
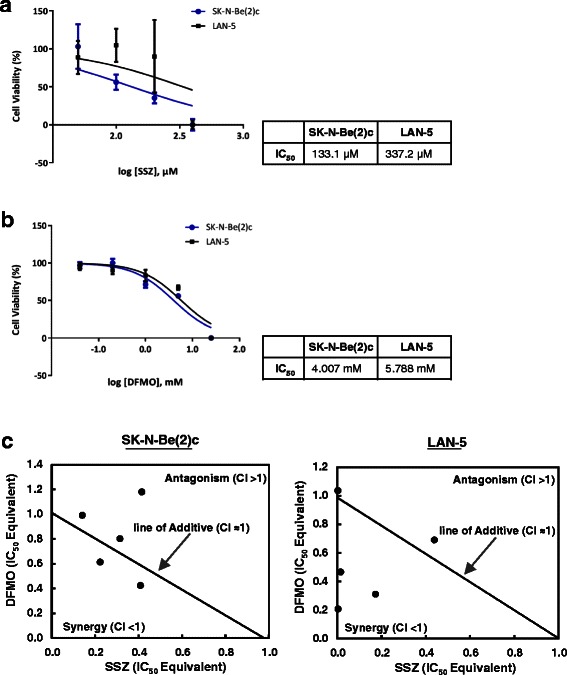
Isobologram analysis for SSZ and DFMO in NB. Isobolograms were prepared to determine synergisms between SSZ and DFMO. NB cell lines SK-N-Be(2)c and LAN-5 were used to determine the inhibitory concentration at which 50 % of cells are dead (IC_50_) after 48 h of treatment with (**a**) SSZ and (**b**) DFMO. (**c**) Isobologram analysis to determine the combined cytotoxicity of SSZ and DFMO using the IC_50_ values from (**a** and **b**). The IC_50_ value of SSZ and DFMO used in combination provides the connective points for the line of additive. Synergy, additivity, or antagonism is indicated below, on, or above the line, respectively. The data present the average of three independent experiments in duplicate (n = 6); points, mean ± SEM

To test whether the combined treatment with SSZ and DFMO induces synergistic cell death in NB, we treated SK-N-Be(2)c and LAN-5 cells with different concentrations of SSZ and DFMO. We used two common methods to analyze drug-drug interactions, the isobologram and the combination index (CI) analysis. For both combination analyses, we measured the SSZ and DFMO interaction at 50 % effect level. We first determined the single-agent IC_50_ concentration for SSZ and DFMO in NB cell lines SK-N-Be(2)c and LAN-5 (Fig. 6, a and b) using an MTS cell viability assay after 48 h of treatment. SSZ exhibited an IC_50_ value of 133.1 μM for SK-N-Be(2)c and 337.2 μM for LAN-5 cells. DFMO showed an IC_50_ value of 4.07 mM for SK-N-Be(2)c and 5.79 mM for LAN-5 cells. Subsequently, we combined SSZ and DFMO at different concentrations based on each IC_50_ value to treat the two NB cell lines, generated isobolograms, and calculated the CI values illustrating the observed synergy. As shown in Fig. 6c and Table 3, SSZ and DFMO combinations revealed slight synergism in SK-N-Be(2)c cells when drug concentrations were below 29.64 μM and 1.80 mM, respectively. Strikingly, SSZ and DFMO showed strong synergism in LAN-5 cells when drug concentrations were below 1.20 μM and 1.21 mM, respectively.

**Table 3:** Combination treatment of SSZ and DFMO in SK-N-Be(2)c and LAN-5 cells for 48 h

	Concentration, IC_50_ Equivalent					
NB Cell Line	SSZ	DFMO	Combination Index at 50 % Effect Level	Evaluation at 50 % Effect Level	SSZ IC_50_ (μM)	DFMO (mM)
**SK-N-Be(2)c**	***0.408***	***0.425***	***0.834***	***slight synergism***	***54.360***	***1.800***
	***0.223***	***0.614***	***0.837***	***slight synergism***	***29.640***	***2.600***
	0.314	0.803	1.117	moderate antagonism	41.740	3.400
	0.140	0.992	1.132	moderate antagonism	18.700	4.200
	0.415	1.181	1.595	antagonism	55.180	5.000
**LAN-5**	***0.004***	***0.207***	***0.211***	***strong synergism***	***1.207***	***1.200***
	***0.173***	***0.311***	***0.484***	***synergism***	***58.250***	***1.800***
	***0.015***	***0.466***	***0.482***	***synergism***	***5.152***	***2.700***
	0.439	0.691	1.130	moderate antagonism	147.900	4.000
	0.003	1.037	1.039	additive	0.893	6.000

**Legend:** The concentration in IC_50_ equivalent of SSZ was calculated by dividing the IC_50_ of SSZ with DFMO combination from its corresponding single-agent IC_50_ value (IC_50_ of SSZ w/ DFMO comb/SSZ IC_50_). For DFMO, the concentration in IC_50_ equivalent was calculated by dividing its actual concentration used in the combination treatment from its corresponding single-agent IC_50_ value (DFMO/ DFMO IC_50_). Combination index (CI) at 50 % effect level is calculated by adding the IC_50_ equivalent concentration of SSZ and DFMO. CI >1.3 is antagonism; CI = 1.1-1.3 is moderate antagonism; CI = 0.9-1.1 is additive; CI = 0.8-0.9 is slight synergism; CI = 0.6-0.8 is moderate synergism; CI = 0.4-0.6 is synergism; CI = 0.2-0.4 is strong synergism. Synergism was detected at two different combinations of DFMO and SSZ in SK-N-Be(2)c cells and three different combinations in LAN-5 cells (*bold italics*). The data present the average of three independent experiments performed in duplicate (n = 6)

## Discussion

SSZ is a salicylate-based anti-inflammatory drug; one of the most important medicines used worldwide in basic health care according to the WHO Model List of Essential Medicines (http://www.who.int/medicines/publications/essentialmedicines/en/). Its mode of action involves the anti-inflammatory and immune-modulatory properties of its metabolic constituent, 5-aminosalicylic acid [31, 36]. SSZ is most commonly used to treat bowel inflammation, diarrhea, rectal bleeding, and abdominal pain in patients with ulcerative colitis. So far, nothing is known about a potential therapeutic effect of SSZ in NB.

Molecular and computational studies presented in this work and in [32] suggest that the SSZ target molecule SPR may constitute a novel druggable protein in NB. Both chains of the SPR homodimer were found to simultaneously bind ligands in the docking simulations and the SSZ binding site was located at the NADP-binding Rossmann fold. Thus, competition between SSZ and NADP+ may modulate or inhibit the activity of SPR as the two ligands do not have an equivalent enzymatic role. In addition to occupying the same receptor pocket, complex formation with SSZ could locally perturb the dimerization interface. Binding region 4 includes the aromatic residue Tyr 167 that is situated near the dimer interface in a relatively apolar area and may affect the thermodynamics of ligand and inhibitor binding as well as the protein dimerization. It remains to be clarified in further work whether the primary physiological role of SSZ is competitive/non-competitive inhibition or perturbation of dimerization which would in turn disrupt the functional biological unit in addition to the enzymatic changes.

## Conclusions

The results of the NB cell experiments show that SSZ has a detrimental effect on NB cells in *in vitro* culture and shows synergy with DFMO treatment which is encouraging. The identification of the molecular pathways that are activated in response to SSZ action will need further studies. Considering the low toxicity of DFMO and its current use in NB clinical trials [26–28], a combination with the equally low toxic and clinically evaluated SSZ appears a good lead for future clinical studies.

## Methods

### Mammalian cell culture and reagents

The human NB cell line SK-N-Be(2)c was obtained from Dr. Giselle Sholler (Helen DeVos Children’s Hospital, Grand Rapids, MI). The human NB cell line LAN-5 was obtained from Dr. Randal Wada (John A. Burns School of Medicine, University of Hawaii at Manoa, Honolulu, HI). The human NB cell line SK-N-SH was purchased from the American Type Culture Collection (Manassas, VA). Cells were maintained in RPMI 1640 media (Mediatech Inc, Manassas, VA) containing 10 % heat-inactivated fetal bovine serum (FBS) (Atlanta Biologicals, Inc, Lawrenceville, GA), penicillin (100 IU/mL), and streptomycin (100 Ag/mL) (Mediatech). Sulfasalazine (SSZ) (Santa Cruz Biotechnology, Inc, Dallas, TX) stock solution was prepared at 250 mM concentration in dimethyl sulfoxide (DMSO) (Electron Microscopy Sciences, Hatfield, PA). DFMO was a kind gift of Dr. Patrick Woster (Medical University of South Carolina, Charleston, SC) and dissolved in water to make a stock solution of 250 mM as previously reported [18, 19, 21]. SSZ and DFMO were diluted with culture medium before treating the cells. An equal concentration of DMSO was used for control treatments.

### Cell viability assay

Prior to treatment, cells were cultured overnight in 96-well microtiter plates (Greiner Bio-One Inc, Monroe, NC). LAN-5, SK-N-Be(2)c, or SK-N-SH cells were seeded at concentrations of 1.5, 5.0, or 1.0 × 10^4^ cells per well, respectively. All NB cell lines were suspended in 90 μl of medium per well. After overnight incubation, NB cells were treated with increasing concentrations of SSZ (0–400 μM) or DFMO (0–25 mM) for 48 h. An equal concentration of DMSO was used as a control. Cell viability was measured with the CellTiter 96 AQueous One Solution Cell Proliferation Assay (MTS Assay) (Promega BioSciences, San Luis Obispo, CA) following the manufacturer’s protocol. Briefly, 20 μL of CellTiter 96 AQueous One Solution Reagent was added to each well and incubated at 37 °C for 3 h. The quantity of formazan product that is proportional to the number of living cells in the culture was measured at 490 nm using the Synergy Mx Monochromator-Based Multi-Mode Microplate Reader (BioTek Instruments, Inc, Winooski, VT). Optical density (OD) readings were calculated and evaluated using Excel spreadsheet software (Microsoft, Redmund, WA).

### Isobologram and combination index analyses

Isobologram and combination index (CI) analyses were performed as previously described [37–40] with some modifications. Isobologram analysis is a graphical presentation of the interaction of two drugs at a chosen effect level, such as 50 % effect level or IC_50_ equivalent concentration. CI analysis is used to quantitatively measure the interaction of two drugs at a chosen effect level. In this study, the 50 % effect level was used for both analyses. The IC_50_ values of SSZ and DFMO for SK-N-Be(2)c and LAN-5 NB cell lines were calculated using the nonlinear log inhibitor versus normalized response curve fit function from GraphPad Prism 6 software (La Jolla, CA). Based on this single-agent IC_50_ determination, each NB cell line was treated with a combination of SSZ and DFMO at different concentrations. Seven different concentrations of SSZ ranging from 2.34 μM to 150 μM, and 5.47 μM to 350 μM were used to treat SK-N-Be(2)c and LAN-5 cells, respectively. Five different concentrations of DFMO ranging from 1.8 mM to 5.0 mM, and 1.2 mM to 6.0 mM were used to treat SK-N-Be(2)c and LAN-5, respectively. The CellTiter 96 AQueous One Solution Cell Proliferation Assay (Promega) was used to measure the drug activity for each NB cell line. Excel spreadsheet software and GraphPad Prism 6 software were used to plot the isobologram and determined the CI for each NB cell line combination treatment. The line of additivity on the isobologram represents the 50 % effect level of each drug.

### Protein–ligand docking

Atomic coordinates from X-ray crystal structures of human sepiapterin reductase (SPR; PDB:1Z6Z) were obtained from the Protein Data Bank [41] and used for molecular docking. The crystallographic assembly is a homo 6-mer (A6) and the single repeating unit consists of residues L(−)5 to K258. The protein chain is in complex with NADP+. The quaternary structure of the biological unit is a homo 2-mer (A2).

Sulfasalazine (Compound ID: 5384001/5359476) structure information was retrieved from the PubChem Substance and Compound Database [35]. Three-dimensional coordinates were available for a stable conformer, energy minimized by the MMFF94 force field [33].

Molecular docking was carried out to locate plausible SSZ binding sites in SPR. The Global Range Molecular Matching method (GRAMM) was employed on local computers in high-resolution geometric docking modes using both a long-distance-potentials approach [42] and correlation techniques [43]. The GRAMM algorithm identifies the docking areas by computing the intermolecular energy potential in protein–ligand complexes through a comprehensive multidimensional search of relative molecular positions and orientations. A low-resolution semi-flexible mode was also used to account for conformational flexibility [44, 45].

The docking simulations were run with SPR monomers and dimers, each in complex with the energy–minimized SSZ conformer. The first 50 binding locations of every run were scored by the binding energy between the ligand and the protein and by the presence or absence of amino acid residues in the contact surfaces among the various protein–ligand pairs. The complexes with the lowest spatial variations were chosen as the most plausible models. The predicted binding sites were visualized with the ICM-Browser (Molsoft, San Diego, CA). The ICM Molecular Editor (Molsoft) was used for chemical structure drawing.

### NB public mRNA expression dataset analysis

Human NB mRNA expression datasets in the public domain were analyzed using R2: a genomics analysis and visualization platform developed in the Department of Oncogenomics at the Academic Medical Center – University of Amsterdam (http://r2.amc.nl). Expression data (CEL files) for the datasets were retrieved from the public Gene Expression Omnibus (GEO) dataset on the NCBI website (http://www.ncbi.nlm.nih.gov/geo/). All analysis of human material and human data was in compliance with the “Declaration of Helsinki for Medical Research involving Human Subjects” (http://www.wma.net/en/30publications/10policies/b3/index.html). In addition, approval was obtained from the “Medisch Ethische Commissie (MEC) van het AMC (Amsterdam)”, the local research and ethics committee. CEL data were analyzed as described in [46]. Briefly, gene transcript levels were determined from data image files using GeneChip operating software (MAS5.0 and GCOS1.0, from Affymetrix). Samples were scaled by setting the average intensity of the middle 96 % of all probe-set signals to a fixed value of 100 for every sample in the dataset, allowing comparisons between micro-arrays. The TranscriptView genomic analysis and visualization tool within R2 was used to check if probe-sets had an anti-sense position in an exon of the gene (http://r2.amc.nl > genome browser). The probe-sets selected for SPR (Affymetrix 203458_at and Illumina 1705849) and ODC1 (Affymetrix 200790_at and Illumina 1748591) meet these criteria. All expression values and other details for the datasets used can be obtained through their GSE number from the NCBI GEO website.

### Statistical analysis

SPR mRNA expression and correlation with important NB clinical parameters were determined using the non-parametric Kruskal-Wallis test; correlation with ODC mRNA expression was calculated with a 2log Pearson test. The significance of a correlation is determined by t = R/sqrt((1-r^2)/(n-2)), where R is the correlation value and n is the number of samples. Distribution measure is approximately as t with n-2° of freedom. For all tests, *P* < 0.05 was considered statistically significant. The statistical significance of SSZ treatments in cell viability experiments was determined by Microsoft Excel’s Student’s paired *t*-Test, with one-tailed distributions.

## Abbreviations

DFMO: alpha-difluoromethylornithine
NADP: Nicotinamide adenine dinucleotide phosphate
SPR: Sepiapterin reductase
SSZ: Sulfasalazine
